# Prone position ameliorates lung elastance and increases functional residual capacity independently from lung recruitment

**DOI:** 10.1186/s40635-015-0055-0

**Published:** 2015-06-11

**Authors:** Alessandro Santini, Alessandro Protti, Thomas Langer, Beatrice Comini, Massimo Monti, Cristina Carin Sparacino, Daniele Dondossola, Luciano Gattinoni

**Affiliations:** Dipartimento di Fisiopatologica Medico-Chirurgica e dei Trapianti, Università degli Studi di Milano, Via Francesco Sforza 35, 20122 Milan, Italy; Dipartimento di Anestesia, Rianimazione ed Emergenza Urgenza, Fondazione IRCCS Ca’ Granda–Ospedale Maggiore Policlinico di Milano, Milan, Italy; Centro di Ricerche Chirurgiche Precliniche, Fondazione IRCCS Ca’ Granda–Ospedale Maggiore Policlinico, Università degli Studi di Milano, Milan, Italy

**Keywords:** Prone position, Supine position, Lung recruitment, Pressure–volume curve, Lung elastance, Functional residual capacity

## Abstract

**Background:**

Prone position is used to recruit collapsed dependent lung regions during severe acute respiratory distress syndrome, improving lung elastance and lung gas content. We hypothesised that, in the absence of recruitment, prone position would not result in any improvement in lung mechanical properties or gas content compared to supine position.

**Methods:**

Ten healthy pigs under general anaesthesia and paralysis underwent a pressure–volume curve of the respiratory system, chest wall and lung in supine and prone positions; the respective elastances were measured. A lung computed tomography (CT) scan was performed in the two positions to compute gas content (i.e. functional residual capacity (FRC)) and the distribution of aeration. Recruitment was defined as a percentage change in non-aerated lung tissue compared to the total lung weight.

**Results:**

Non-aerated (recruitable) lung tissue was a small percentage of the total lung tissue weight in both positions (4 ± 3 vs 1 ± 1 %, supine vs prone, *p* = 0.004). Lung elastance decreased (20.5 ± 1.8 vs 15.5 ± 1.6 cmH_2_O/l, supine vs prone, *p* < 0.001) and functional residual capacity increased (380 ± 82 vs 459 ± 60 ml, supine vs prone, *p* = 0.025) in prone position; specific lung elastance did not change (7.0 ± 0.5 vs 6.5 ± 0.5 cmH_2_O, supine vs prone, *p* = 0.24). Lung recruitment was low (3 ± 2 %) and was not correlated to increases in functional residual capacity (*R*^2^ 0.2, *p* = 0.19). A higher amount of well-aerated and a lower amount of poorly aerated lung tissue were found in prone position.

**Conclusions:**

In healthy pigs, prone position ameliorates lung mechanical properties and increases functional residual capacity independently from lung recruitment, through a redistribution of lung aeration.

## Background

Prone position is used as a rescue therapy during acute respiratory distress syndrome (ARDS) in severely hypoxic patients [1, 2] in whom it usually improves oxygenation and lung mechanics [3, 4]. The oxygenation benefit is due to a better ventilation–perfusion matching [5, 6] and/or to recruitment of dorsal lung parenchyma with a decrease in shunt fraction [4, 7, 8]. The change in the mechanical properties of the lung is usually attributed to lung recruitment with an increase in lung resting volume [3, 9] and to a lower vertical pleural pressure gradient in prone position, with consequent more homogenous distribution of transpulmonary pressure, lung inflation and thus ventilation [5, 6, 10].

Since the first description of ARDS, it became evident that mechanical ventilation per se can worsen lung damage, spread systemic inflammation and affect outcome [1] and a new nosologic entity was defined, namely ventilator-induced lung injury (VILI). Many animal studies either on healthy or diseased lungs have reported the role of prone position in delaying VILI appearance [11, 12] or in reducing its severity [13–15].

The determinants of VILI are excessive pressures acting on lung parenchyma (i.e. transpulmonary pressure or stress) and lung deformation over lung resting volume (i.e. tidal volume/functional residual capacity or strain), closely linked to each other by lung intrinsic mechanical properties (i.e. specific lung elastance) [16, 17]. The protective effect of prone position could accordingly be due either to an increase in lung resting volume (i.e. functional residual capacity (FRC)) with a consequent decrease in strain and transpulmonary pressure for the same tidal volume applied or to the prevention of opening and closing of lung units during tidal ventilation (atelectrauma).

Both mechanisms of protection (increase in FRC with decrease in transpulmonary pressure and reduction of atelectrauma) are usually thought to depend on the presence of recruitable lung tissue, i.e. non-aerated, collapsed lung units which undergo reopening and aeration during the respiratory cycle [18], and should have, if any, a marginal role in healthy lungs or in diseased lungs with low recruitable tissue.

In this study, we investigated lung mechanical properties and volumes in supine and prone positions in a healthy animal model, with a small amount of non-aerated, potentially recruitable lung tissue. Our hypothesis was that, in the absence of potential for lung recruitment, prone position would not result in any change in lung mechanical behaviour and lung resting volume compared to supine position. In contrast with our hypothesis, we found that even when recruitment is negligible, prone position ameliorates lung elastance and increases functional residual capacity, mainly through a different, more homogenous distribution of lung aeration.

## Methods

The study was approved by the Institutional Review Board and was conducted in accordance to international recommendations on animal care [19].

Ten healthy piglets weighing 21 ± 2 kg were studied. Anaesthesia induction and surgical preparation were performed as previously described, in supine position [16]. A 5-cm-long latex oesophageal balloon was placed in the lower third of the oesophagus to estimate changes in pleural pressure. Proper positioning was confirmed with computed tomography (CT) scan (see below).

Each animal was studied both in supine and prone positions, after a recruitment manoeuvre (1 min of pressure-controlled ventilation with inspiratory pressure 40 cmH_2_O, PEEP 5 cmH_2_O, I:E = 0.5, F_i_O_2_ = 0.5). A static pressure (airway and oesophageal)–volume curve was recorded with a dedicated software (Colligo, www.elekton.it). A 100-ml calibrated glass syringe was attached to the endotracheal tube, and 100-ml aliquots of room air were inflated into the lungs until an airway pressure of 35 cmH_2_O was reached. Each step lasted 5 s, reaching static conditions.

From airway (*P*_aw_) and oesophageal (*P*_es_) pressure recordings, transpulmonary pressure (*P*_L_) was calculated as:

1 $$ {P}_{\mathrm{L}} = \left({P}_{\mathrm{aw}} - {P}_{\mathrm{aw},\mathrm{ZEEP}}\right) - \left({P}_{\mathrm{es},\mathrm{vol}} - {P}_{\mathrm{es},\mathrm{ZEEP}}\right) $$

where *P*_aw_ is the airway pressure, *P*_aw,ZEEP_ is the airway pressure at end expiration (verified to be always equal to the atmospheric pressure), *P*_es,vol_ is the oesophageal pressure at any given inflation volume and *P*_es,ZEEP_ is the oesophageal pressure at end expiration.

For each pig, the respiratory system (*P*_aw_–volume), chest wall (*P*_es_–volume) and lung (*P*_L_–volume) pressure–volume curves in prone and supine positions were computed. The slope of the linear part (by visual inspection) of each pressure–volume curve was used to measure the respective elastance value. Once functional residual capacity was measured (see below), specific lung elastance was computed as the slope of the linear part (by visual inspection) of the stress–strain curve.

After an additional recruitment manoeuvre, a lung CT scan was performed at end expiration with the endotracheal tube clamped, in prone and supine positions. CT scan settings have been described elsewhere [16, 20], and lung CT scan-derived lung weight measurement has been previously validated in this same animal model by our group [21].

Lung CT images were manually outlined including only lung parenchyma and excluding bronchi and big intrapulmonary vessels and analysed with a dedicated software (Maluna 3.17, University Hospital of Göttingen, Germany) to measure lung gas content (i.e. functional residual capacity), lung weight and aeration distribution, as previously described [22]. Lung tissue was divided into four compartments based on the degree of aeration: non-aerated tissue (density from 0 to −100 H.U.), poorly aerated tissue (density from −101 to −500 H.U.), well-aerated tissue (density from −501 to −900 H.U.) and over-aerated tissue (density from −901 to −1000 H.U.). Lung tissue weight of each compartment was expressed as a percentage of the total lung weight. We defined recruitment as:

2 $$ \begin{array}{l}\mathrm{Lung}\ \mathrm{recruitment}\ \left(\%\right) = \Big(\mathrm{n}\mathrm{o}\mathrm{n}\hbox{-} \mathrm{aerated}\ \mathrm{lung}\ \mathrm{tissue}\ \mathrm{weight}\ \left(\mathrm{g}\right)\ \mathrm{in}\ \mathrm{supine}\ \mathrm{position} - \mathrm{n}\mathrm{o}\mathrm{n}\hbox{-} \mathrm{aerated}\ \mathrm{lung}\ \mathrm{tissue}\ \mathrm{weight}\\ {}\ \left(\mathrm{g}\right)\ \mathrm{in}\ \mathrm{prone}\ \mathrm{position}\Big)/\mathrm{total}\ \mathrm{lung}\ \mathrm{weight}\ \left(\mathrm{g}\right)\ \mathrm{in}\ \mathrm{supine}\ \mathrm{position}\times 100\end{array} $$

The coefficient of variation of voxel density distribution, used as a surrogate measure of homogeneity of aeration distribution, was calculated as the standard deviation of CT densities divided by mean CT density in each position.

### Statistical analysis

Data are reported as mean ± standard deviation unless otherwise stated. Variables acquired in the two positions were compared with paired Student’s *t* test. The change in weight of each lung compartment (non-aerated, poorly aerated, well-aerated and over-aerated) between the two positions is always expressed as a percentage change compared to the total lung weight. Linear regression was used to correlate changes in normally distributed variables between the two positions.

Statistical analysis was performed with Stata software (StataCorp. 2013. Stata Statistical Software: Release 13. College Station, TX: StataCorp LP.).

## Results

Ten healthy pigs were studied in supine and prone positions. Non-aerated, potentially recruitable lung tissue weight was less than 5 % of the total lung weight in both positions, and lung recruitment was negligible (3 ± 2 % passing from supine to prone position).

**Fig. 1:**
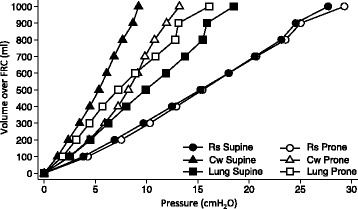
Pressure–volume curves of respiratory system, chest wall and lung in supine and prone positions. Mean respiratory system (*Rs*; *circles*), chest wall (*Cw*; *triangles*) and lung (*squares*) pressure–volume curves in supine (*black*) and prone (*white*) positions. Starting from functional residual capacity (*FRC*; volume = 0 ml), volume was inflated in 100-ml aliquots. After 5 s, the corresponding airway, oesophageal or transpulmonary pressure was recorded. Each symbol represents the mean value (*n* = 10), and standard deviations are not shown for clarity

Mean respiratory system elastance was not different in the two positions. However, chest wall elastance worsened (10.6 ± 1.0 vs 17.0 ± 0.7 cmH_2_O/l, supine vs prone, *p* < 0.001), and lung elastance ameliorated (20.5 ± 1.8 vs 15.5 ± 1.6 cmH_2_O/l, supine vs prone, *p* < 0.001) in prone position (Fig. 1).

**Fig. 2:**
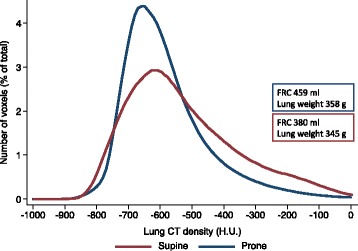
Frequency distribution of voxels in supine and prone positions. Mean number of voxels, expressed as a percentage of the total number of voxels, for every 10 H.U. changes in CT-derived lung density, in supine (*red*) and prone (*blue*) positions. The *red* and *blue inserts* refer to the mean values of *FRC* and lung weight in supine and prone positions, respectively. The coefficient of variation of voxel distribution was 0.32 in supine and 0.23 in prone positions

A higher gas content, i.e. functional residual capacity, was found (380 ± 82 vs 459 ± 60 ml, supine vs prone, *p* = 0.025), in spite of a similar amount of aerated lung tissue, due to a different distribution of gas in the alveoli in prone compared to supine position (Table 1). A more homogeneous distribution of aeration in prone position is indicated by the lower coefficient of variation of voxel density distribution (Fig. 2).

**Table 1:** Lung volume, weight and distribution of aeration in supine and prone positions

	Supine	Prone	*p* value
Total lung weight (g)	345 ± 26	358 ± 44	0.420
Aerated lung weight (g)	330 ± 24	353 ± 43	0.156
Functional residual capacity (ml)	380 ± 82	459 ± 60	0.025
Non-aerated lung tissue (% of total)	4 ± 3	1 ± 1	0.004
Poorly aerated lung tissue (% of total)	47 ± 13	30 ± 9	0.005
Well-aerated lung tissue (% of total)	49 ± 15	68 ± 10	0.003
Over-aerated lung tissue (% of total)	0 ± 0	0 ± 0	0.432

Lung CT scan-derived values: lung volume, lung tissue weight and lung tissue compartments in prone and supine positions. Aerated lung weight was defined as lung weight having a density <−100 H.U. Lung tissue compartments were divided based on their densities (see text), and the weight of each compartment was expressed as a percentage of the total lung weight. The difference between non-aerated lung tissue weights in the two positions expressed as a percentage of the total lung weight corresponds to lung recruitment (see text)

The change in non-aerated lung tissue weight, i.e. lung recruitment, passing from supine to prone position did not correlate with the observed increase in FRC (*R*^2^ 0.2, *p* = 0.19). The change in poorly aerated lung tissue weight, instead, correlated inversely with FRC change (*R*^2^ 0.41, *p* = 0.046) while the change in well-aerated lung tissue weight correlated directly with FRC change (*R*^2^ 0.53, *p* = 0.02).

**Fig. 3:**
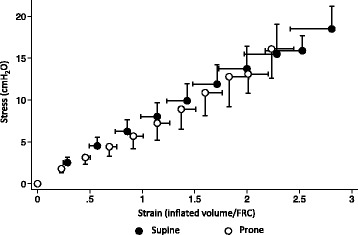
Stress–strain curve in supine and prone positions. Mean and standard error bars (*n* = 10) of the stress–strain curve in supine (*black*) and prone (*white*) positions. Strain was calculated as volume over FRC/FRC; stress was equal to transpulmonary pressure [*P*_L_ = (*P*_aw_ − *P*_aw,ZEEP_) − (*P*_es,vol_ − *P*_es,ZEEP_)] (see text). Each *dot* represents a 100-ml step of lung inflation; pressure was recorded after static conditions were reached. The slope of the linear part of the stress–strain curve, which corresponds to specific lung elastance, is not different between prone and supine positions

Specific (intrinsic) lung elastance was not different (7.0 ± 0.5 vs 6.5 ± 0.5 cmH_2_O, supine vs prone, *p* = 0.24), as shown by the similar slope of the mean stress–strain curve in the two positions (Fig. 3).

## Discussion

In this healthy animal model, the changes in lung mechanics, lung volume and distribution of aeration exclusively due to a change in body position—prone vs supine—were studied. In contrast with our hypothesis, we found a change in lung elastance and functional residual capacity even in the absence of lung recruitment.

Lung mechanical properties improved in prone position, as shown by the different slopes of the lung pressure–volume curve, corresponding to a decrease in lung elastance. An expected worsening in chest wall mechanical properties, i.e. an increase in chest wall elastance, of approximately the same magnitude, paralleled this effect. These two opposite behaviours summed to give a net negligible result on respiratory system elastance. Thus, if partitioning of respiratory system mechanics in lung and chest wall components had not been performed, it would have been impossible to appreciate any change between supine and prone positions (Fig. 1).

This unexpected change in lung mechanical properties in a healthy animal model led us to consider the possibility that functional residual capacity had increased in prone position and to look for lung recruitment as a possible explanation for this increase in lung gas content. Lung recruitment however depends on the amount of non-aerated lung tissue (i.e. on the number of potentially recruitable lung units) [23], which should be low to nil in healthy lungs. In order to clarify whether lung recruitment had occurred, lung CT scans were performed and lung weight, gas content and distribution of aeration were measured.

Functional residual capacity was actually higher in prone than in supine position. The amount of non-aerated lung tissue was a small percentage of the total lung tissue weight, as expected in healthy lungs, and even if changing position from supine to prone resulted in modest lung recruitment, this did not correlate with functional residual capacity change. Altogether, these results make very unlikely that the opening of previously collapsed lung units, corresponding to lung CT scan analysis of non-aerated lung tissue (≥−100 H.U.) which becomes aerated (<−100 H.U.), substantially accounted for the observed increase in FRC. Instead, the opposite changes in poorly and well-aerated lung tissue showed a significant correlation with functional residual capacity change. As the total mass of aerated lung tissue did not change, these density modifications anatomically correspond to already open lung units that get more inflated in prone position (poorly aerated lung tissue which becomes well aerated). This might be considered as a different kind of “recruitment”, which we could define as a redistribution of aeration, i.e. a large amount of lung tissue with poor aeration in supine position, which becomes well aerated in prone position. As with recruitment, when redistribution of aeration occurs, lung gas content increases, but differently from recruitment, the total amount of lung tissue open to aeration does not change. The differences between these two phenomena merit some further explanation: when previously collapsed lung units reopen, the same amount of gas distributes to a higher number of lung units (a larger lung open to aeration), giving as net effect a decrease in pressure. This would typically be seen on the pressure–volume curve as a flat step (an increase in volume without an increase in pressure), which clearly did not happen in our animals, since the lung pressure–volume curves in supine and prone positions never get close to each other.

The mechanical advantage given by the redistribution of aeration instead is probably related to other phenomena, since the total number of lung units open to aeration (i.e. the dimensions of the lung open to aeration) does not change. These might be an increase in the radius of the alveoli open to aeration, which lowers the elastance of each alveolus, and a higher homogeneity in the distribution of aeration (see Fig. 2), which through interdependence affects the elastance of higher radius alveoli close to lower radius alveoli.

We do not know whether the major contributor to lung mechanical improvement is the increase in volume itself, or the “quality” of the lung tissue open to aeration, since well-aerated lung tissue, not poorly aerated, has been associated to the mechanical properties of the lung in patients with ARDS [22]. We however propose redistribution of aeration as a mechanism, different from classically defined lung recruitment, which could explain an amelioration of lung elastance and an increase in lung gas content associated with prone positioning, even in those situations in which a very low amount of non-aerated recruitable lung tissue is present and recruitment is expected to be low.

## Conclusions

In this healthy animal model in which lung recruitment was negligible, prone position was associated with an amelioration of lung mechanics, a higher functional residual capacity and a different, more homogenous distribution of lung aeration than supine position. Lung recruitment, defined as an opening of previously collapsed lung units, is thus not necessary per se for prone position to improve lung mechanical properties and increase lung gas content.

## Abbreviations

ARDS: acute respiratory distress syndrome
CT: computed tomography
FRC: functional residual capacity
H.U.: Hounsfield units
*P*_aw_: airway pressure
*P*_es_: oesophageal pressure
*P*_L_: transpulmonary pressure
VILI: ventilator-induced lung injury
